# Endometrial cancer in Puerto Rico: incidence, mortality and survival (1992-2003)

**DOI:** 10.1186/1471-2407-10-31

**Published:** 2010-02-03

**Authors:** Ana Patricia Ortiz, Javier Pérez, Yomayra Otero-Domínguez, Omar García-Rodríguez, Sheyla Garced-Tirado, Frances Escalera-Maldonado, Sadja Gaud-Quintana, Elvis Santiago-Rodríguez, Katherine Svensson, José L Vergara-Arroyo, Karen Ortiz, Mariela Torres, Guillermo Tortolero-Luna, Nayda Figueroa-Vallés

**Affiliations:** 1 Cancer Control and Population Sciences Program, University of Puerto Rico Comprehensive Cancer Center, San Juan, Puerto Rico; 2 Department of Biostatistics and Epidemiology, Graduate School of Public Health, Medical Sciences Campus, University of Puerto Rico, San Juan, Puerto Rico; 3 Puerto Rico Central Cancer Registry, Cancer Control and Population Sciences Program, University of Puerto Rico Comprehensive Cancer Center, San Juan, Puerto Rico

## Abstract

**Background:**

Endometrial cancer is the most common gynecologic malignancy in Puerto Rico and the United States (US).

**Methods:**

We compare the age-specific and age-adjusted incidence and mortality rates and the survival of endometrial cancer in Puerto Rico with that of non-Hispanic whites (NHW), non-Hispanic blacks (NHB) and Hispanics in the US. Data from the Puerto Rico Central Cancer Registry and the Surveillance, Epidemiology, and End Results program were analyzed from 1992-2003.

**Results:**

Age-standardized incidence rates of endometrial cancer increased significantly (p < 0.05) in Puerto Rico (APC = 2.8%) and among NHB (APC = 1.9%) and remained constant (p > 0.05) for NHW (APC = -0.1%) and Hispanics in the US (APC = 0.4%). Mortality trends remained constant in all racial/ethnic groups (p > 0.05). For 1999-2003, women in Puerto Rico had similar incidence of endometrial cancer as Hispanics (Standardized rate ratio [SRR] = 0.94, 95% CI = 0.87-1.01), although their risk was lower than that of NHW (SRR = 0.56, 95% CI = 0.53-0.59) and NHB (SRR = 0.91, 95% CI = 0.84-0.98). Meanwhile, women in Puerto Rico had 15% higher risk of death than Hispanic women (SRR = 1.15, 95% CI = 1.03-1.30) similar risk than NHW (SRR = 0.93, 95% CI = 0.83-1.03), and lower risk than NHB (SRR = 0.51, 95% CI = 0.46-0.57). Puerto Rico (63.1%) and NHB (56.8%) had a lower 5-year survival than NHW (78.4%) and Hispanics (79.5%). An age-adjusted Cox proportional hazards model showed that compared with women in Puerto Rico, Hispanic women in the United States had 37% lower mortality risk (HR = 0.63, 95% CI = 0.56-0.71) and NHW had 53% lower mortality risk (HR = 0.47, 95% CI = 0.43-0.52) after 5 years of diagnosis; NHB women had 22% higher mortality risk than women in Puerto Rico (HR = 1.22, 95% CI = 1.09-1.36).

**Conclusions:**

The lower burden of endometrial cancer in Puerto Rico suggests the presence of protective factors or lower exposure to risk factors in this population, although increases in incidence suggest changes in the occurrence of lifestyles and environmental risk factors. Meanwhile, the lower five-year survival from endometrial cancer among Puerto Ricans suggests a health disparity for this group in areas such as quality of care and/or differences in terms of stage at diagnosis and associated comorbidities. Assessment of disease risk factors and characteristics, and access and response to treatment is required to further understand these results.

## Background

Uterine cancer is the most common gynecologic malignancy in Puerto Rico and the United States. In the United States, uterine cancer is the fourth most common cancer in women, accounting for approximately 6% of all cancers [1,2]. In Puerto Rico, uterine cancer is the third most common cancer type, also representing approximately 6% of all cancers [3]. Despite the importance of this malignancy among Puerto Rican women, there is no recently published data on the burden of endometrial cancer in Puerto Rico. Previous studies in the 70's and 80's showed a lower incidence of the disease among women living in Puerto Rico as compared to the general population in the United States [4] and to Puerto Ricans [5] living in the continental United States.

Most literature on ethnic differences of cancer of the corpus uterus in the United States has focused mainly on non-Hispanic whites (NHW) and non-Hispanic Blacks (NHB), with fewer comparisons with other racial/ethnic groups, such as Hispanics [6]. Although Puerto Rico is a Commonwealth territory of the United States since 1898, cancer data for Puerto Rico is not currently included in the United States cancer statistics [7]. Knowledge of the burden of cancer in specific populations is important to identify health disparities among populations [7]. To further our understanding of the burden of endometrial cancer in Puerto Rico and how it compares with other racial/ethnic groups in the United States, we compare the age-specific and age-adjusted incidence and mortality rates and the survival of endometrial cancer in Puerto Rico with that of Hispanics, NHW, and NHB in the United States for the period 1992-2003, years in which the National Cancer Institute's Surveillance, Epidemiology, and End Results program [SEER] first began coding Hispanic ethnicity.

## Methods

### Data Sources

Incident data of endometrial cancer for Puerto Rico were obtained from the incidence case file [8] from 1992-2003. The Puerto Rico Central Cancer Registry is the fourth oldest population-based cancer registry in the world [9] collecting information in Puerto Rico since 1951. Since 1997, the Puerto Rico Central Cancer Registry has been part of the CDC's National Program of Cancer Registry and uses the SEER and the North American Association of Central Cancer Registries (NAACCR) standards for coding data; thus, the registry is fully comparable with both SEER and NAACCR data. In the year 2003, a CDC audit concluded that 95.3% of all cancer cases diagnosed or treated in hospital facilities in Puerto Rico were appropriately reported to the Puerto Rico Central Cancer Registry; a result comparable to the United States median (95%) [10]. All cancer cases diagnosed since 2001 are reported using the third edition of the International Classification of Disease for Oncology (ICD-O-3) [11]. Cases from 1992 to 2000 which were originally reported using previous editions of ICD-O were converted to ICD-O-3 codes. Incident cases of endometrial cancer for NHW, NHB, and Hispanics in the United States for the period from 1992 to 2003 were obtained from the SEER 13 [SEER Stat 6.3.5 software] [12].The database SEER 13 represents 13.8% of the total US population; the Hispanic category represents 21.8% of the total US Hispanic population and includes persons from multiple Hispanic sub-groups (i.e. Mexicans, Puerto Ricans, Cubans, among others living in the US). Incidence data for Hispanics are based on the NAACCR Hispanic Identification Algorithm (NHIA) [13].

Endometrial cancer mortality information for Puerto Rico and the United States (NHW, NHB, Hispanics) from 1992-2003 was obtained, respectively, from the Puerto Rico Central Cancer Registry as reported by death certificates prepared by the Puerto Rico Department of Health [14] and from the SEER program as reported by the National Center for Health Statistics (NCHS) [15]. Causes of death were coded and classified according to the International Classification of Diseases (ICD-10) [16].

Women diagnosed with a primary cancer of the endometrium (ICD-O3 C54.1 with histological valid codes from 8000-8576 or by death certificate from cancer associated to the endometrium or corpus and uterus, not otherwise specified (NOS) (ICD-10 C54-C55; ICD-9 179, 182)), between 1992 and 2003 were eligible for our study. For incidence statistics, the analysis was limited to primary cases of malignant endometrial cancer confirmed microscopically, with a positive histology, positive exfoliative cytology with no positive histology, a positive laboratory test/marker study or direct visualization without microscopic confirmation, radiography without microscopic confirmation, clinical diagnosis only or those diagnosed solely by death certificates. Cases of carcinoma in situ and uterine sarcomas were excluded. For mortality statistics, cases with cause of death stated as corpus and uterus, NOS were eligible for analysis. Mortality from endometrial cancer was evaluated combining corpus and NOS categories as these categories are usually combined to reflect non-cervical carcinoma of the uterus and given that cases diagnosed through death certificates are more likely to have NOS classification. Data quality across all sub-groups was good, as the proportion of death certificate only cases was low across all racial/ethnic groups (<1%) (data not shown).

### Statistical Analysis

#### Incidence and mortality rates

We calculated the age-specific annual incidence and mortality rates of endometrial cancer (per 100,000 women) and their 95% confidence intervals (CI) for women aged 20 years and older for Puerto Rico and the following mutually exclusive racial/ethnic groups in the United States: NHW, NHB and Hispanics. Age-specific incidence rates by racial/ethnic group were calculated by dividing the annual number of cases in each age and racial/ethnic group by the estimated annual total population of women in the same age and racial/ethnic group. Age-specific mortality rates in each racial/ethnic group were calculated similarly using age-specific cases of endometrial cancer deaths and the number of women who died from other causes in each age group. Age-specific incidence and mortality rates in each racial/ethnic group were then age-standardized by the direct method to the 2000 Standard Population of the United States, provided by Census Bureau [17,18]. The annual percent change (APC) was estimated to evaluate overall and age-specific incidence and mortality trends of endometrial cancer from 1992 to 2003 using SEER*Stat Software and Joinpoint Regression software program 3.0 [19]. This calculation involved fitting a least squares regression line to the natural logarithm of the rates in each ethnic group, using the calendar year as a regressor variable.

To assess racial/ethnic group differences, the incidence and mortality rates were grouped from 1999-2003. Then, the ratio of two standardized rates was  estimated with its 95% confidence interval [20], to assess significant differences in endometrial cancer between persons living in Puerto Rico and Hispanics, NHW and NHB in the United States, using the STATA System release 10.0 (STATA Corp, College Station, TX, United States). These ratios were denoted as incidence and mortality standardized rate ratios (SRR).

#### Survival

The 5-year observed survival curves for women aged 20 years and older were calculated from 1992-2002 for each racial/ethnic group using the incidence case files of Puerto Rico and the SEER 17. Survival curves by racial/ethnic group were estimated using the Kaplan-Meier method [21] and differences between survival curves were assessed using the log-rank test [22], using SAS 9.1. Cases lost to follow up and those alive at the end of the follow-up period (December 31, 2004) were considered censored observations. In addition, cases with a death after the 5-year follow-up interval were considered alive at the end of those 5 years. A Cox-proportional hazards model was used to compare the 5-year endometrial cancer survival by racial/ethnic groups while adjusting for age at diagnosis [21,22].

## Results

### Incidence and mortality rates

From 1992 to 2003, the age-standardized incidence rates of endometrial cancer in Puerto Rico for women aged 20 years and older increased significantly among Puerto Ricans (14.3 in 1992 to 19.5 in 2003) and NHB (16.6 in 1992 to 21.1 in 2003); an average increase of 2.8% per year in Puerto Rico and 1.9% per year in NHB (Table 1). Rates remained relatively constant for NHW (32.2 in 1992 to 32.1 in 2003) and Hispanics (18.9 in 1992 to 20.7 in 2003) in the United States; with non-significant changes in the APCs in these groups (p > 0.05). Women in Puerto Rico, aged 20 to 34 years, (APC = 9.2%) showed the fastest increase in incidence, from 0.2 per 100,000 in 1992 to 3.0 per 100,000 in 2003 (p < 0.05). Increases in incidence were also observed among NHW (APC = 6.4%), Hispanics (APC = 4.4%) and NHB (APC = 10.5%) women aged 20-34 years, although these were not statistically significant among Hispanics in the United States. The number of cases for this age group was relatively small (cases fluctuated from 1 to 13 for PR, from 2 to 11 for NHB, from 9 to 23 for Hispanics and from 12 to 25 among NHW), thus, the APC need careful interpretation. Meanwhile, in all racial/ethnic groups, overall mortality rates for endometrial cancer remained constant from 1992-2003 (p > 0.05) (Table 2). In age stratified analysis, the only significant decreases in endometrial cancer mortality were observed among NHW women aged 65-79 years (APC = -0.7%) and those aged 80+ (APC = -0.6%) (p < 0.05).

**Table 1:** Age-adjusted and age-specific incidence rates and trends from endometrial cancer by racial/ethnic group, 1992-2003^a^

	Puerto Rico	NHW	NHB	Hispanics
**Year: 1992**				
Age-adjusted rate × 100,000				
(95% CI)^a, b^	14.3 (12.2-16.7)	32.2 (31.0-33.4)	16.6 (14.2-19.3)	18.9 (16.4-21.7)
Age-specific rate (95% CI)				
20-34 years	0.2 (0.0-1.3)	0.5 (0.2-0.8)	0.4 (0.0-1.4)	1.5 (0.8-2.6)
35-49 years	6.4 (4.1-9.6)	10.7 (9.5-12.0)	4.5 (2.7-7.1)	10.6 (8.0-13.8)
50-64 years	28.3 (22.0-35.8)	59.6 (55.8-63.5)	22.3 (16.4-29.6)	29.8 (23.3-37.5)
65-79 years	37.2 (28.1-48.4)	101.7 (96.4-107.3)	61.7 (49.2-76.4)	55.7 (43.4-70.4)
80+ years	31.1 (17.0-52.1)	67.0 (60.1-74.4)	50.9 (31.1-78.6)	26.3 (12.0-49.9)
**Year: 2003**				
Age-adjusted rate × 100,000				
(95% CI)^a, b^	19.5 (17.3-21.9)	32.1 (31.0-33.2)	21.1 (18.7-23.7)	20.7 (18.6-22.9)
Age-specific rate (95% CI)				
20-34 years	3.0 (1.6-5.2)	1.2 (0.8-1.8)	0.8 (0.2-2.0)	1.9 (1.2-2.9)
35-49 years	9.3 (6.6-12.7)	12.9 (11.6-14.4)	7.5 (5.3-10.2)	11.0 (8.9-13.6)
50-64 years	38.3 (31.9-45.5)	63.2 (59.9-66.7)	35.4 (29.2-42.6)	40.3 (34.3-46.9)
65-79 years	49.6 (40.2-60.6)	88.2 (82.9.5-93.7)	71.0 (58.1-85.9)	43.1 (34.1-53.7)
80+ years	27.9 (16.8-43.6)	61.4 (55.5-67.8)	35.3 (21.6-53.6)	46.1 (30.4 -67.0)
**Period: 1992-2003**				
Age-adjusted trend (APC %)^b^	2.8*	-0.1	1.9*	0.4
Age-specific trend (APC %)				
20-34 years	9.2*	6.4*	10.5*	4.4
35-49 years	1.4	1.0	5.6*	2.4*
50-64 years	2.8*	0.5	2.2*	1.9*
65-79 years	3.3*	-1.4*	1.5	-2.6*
80+ years	0.8	-3.8*^	-2.6*	0.1

^a ^Annual rates presented for selected years; ^b ^Standardized to the United States 2000 Standard population using the direct method; *p-value < 0.05; ^† ^Not enough cases to make estimate as no cases were reported for some years; ^Period 1992-1997 the APC = 4.1* and 1997-2003 the APC = -3.8*.

**Table 2:** Age-adjusted and age-specific mortality^a ^rates and trends from endometrial cancer by racial/ethnic group, 1992-2003^b^

	Puerto Rico	NHW	NHB	Hispanics
**Year: 1992**				
Age-adjusted rate × 100,000				
(95% CI)^b, c^	5.5 (4.2-7.1)	5.7 (5.5-5.8)	10.0 (9.4-10.7)	4.3 (3.7-4.9)
Age-specific rate (95% CI)				
20-34 years	0.7 (0.2-2.2)	0.1 (0.0-0.1)	0.3 (0.1-0.5)	0.1 (0.0-0.3)
35-49 years	1.1 (0.3-2.9)	0.8 (0.7-0.9)	1.2 (0.8-1.6)	1.2 (0.8-1.8)
50-64 years	4.5 (2.2-8.1)	5.9 (5.5-6.5)	11.4 (9.9-13.1)	5.4 (4.1-7.0)
65-79 years	18.6 (12.4-26.9)	20.2 (19.4-21.0)	38.1 (34.7-41.8)	13.7 (10.8-17.1)
80+ years	31.1 (17.0-52.2)	32.2 (30.6-33.9)	46.7 (40.0-54.2)	19.4 (13.1-27.7)
**Year: 2003**				
Age-adjusted rate × 100,000				
(95% CI)^b, c^	6.9 (5.6-8.4)	5.6 (5.4-5.7)	10.0 (9.4-10.6)	4.2 (3.7-4.7)
Age-specific rate (95% CI)				
20-34 years	0.2 (0.0-1.3)	0.1 (0.0-0.1)	0.2 (0.1-0.4)	0.1 (0.0-0.2)
35-49 years	2.7 (1.3-4.8)	0.9 (0.8-1.0)	1.3 (1.0-1.7)	1.0 (0.7-1.4)
50-64 years	6.8 (4.3-10.2)	6.6 (6.3-7.0)	11.5 (10.2-12.9)	4.6 (3.7-5.7)
65-79 years	21.8 (15.7-29.4)	19.0 (18.2-19.8)	38.0 (34.7-41.4)	13.1 (10.9-15.7)
80+ years	35.3 (22.6-52.5)	29.6 (28.2-31.1)	45.4 (39.7-51.7)	24.5 (19.0-31.0)
**Period: 1992-2003**				
Age-adjusted trend (APC %)^c^	-1.5	-0.1	0.1	0.1
Age-specific trend (APC %)				
20-34 years	†	†	†	†
35-49 years	-3.6	2.1*	1.3	-1.0
50-64 years	0.1	0.9*	-0.5	-0.4
65-79 years	-1.1	-0.7*	0.2	-0.1
80+ years	-1.4	-0.6*	-0.1	1.6

^a ^Mortality statistics were based on Corpus and Uterus, NOS Cancer; ^b ^Annual rates presented for selected years; ^c ^Standardized to the United States 2000 Standard population using the direct method; *p-value < 0.05;

^† ^Not enough cases to make estimate as no cases were reported for some years; ^Period 1992-1997 the APC = 4.1* and 1997-2003 the APC = -3.8*.

For 1999-2003, the SRR showed that women in Puerto Rico had similar risk of endometrial cancer as compared to Hispanics in the United States (SRR = 0.94, 95% CI = 0.87-1.01), although lower risk than that of NHW (SRR = 0.56, 95% CI = 0.53-0.59) and NHB (SRR = 0.91, 95% CI = 0.84-0.98) in the United States. Regarding mortality, the SRR showed that women in Puerto Rico had 15% higher risk of death from endometrial cancer than Hispanic women (SRR = 1.15, 95% CI = 1.03-1.30), similar risk than NHW (SRR = 0.93, 95% CI = 0.83-1.03), and lower risk than NHB (SRR = 0.51, 95% CI = 0.46-0.57) (Table 3).

**Table 3:** Age-standardized incidence and mortality rates (per 100,000) for endometrial cancer during 1999-2003

	Age Standardized Rate (ASR)				Standardized Relative Ratio*(SRR^a^)		
	**Puerto Rico**	**Hispanics**	**NHW**	**NHB**	**Puerto Rico** **vs. Hispanics^b^**	**Puerto Rico** **vs. NHW^b^**	**Puerto Rico** **vs. NHB^b^**
**Incidence**	18.46	19.62	33.13	20.28	0.94 (0.87-1.01)	0.56 (0.53-0.59)*	0.91 (0.84-0.98)*
**Mortality**	5.13	4.43	5.53	10.04	1.15 (1.03-1.30)*	0.93 (0.83-1.03)	0.51 (0.46-0.57)*

^a ^The ratio of two ASR (US 2000) with 95% confidence interval between parentheses; ^b ^Reference group; * Statistically significant.

### 5-year survival

**Figure 1:**
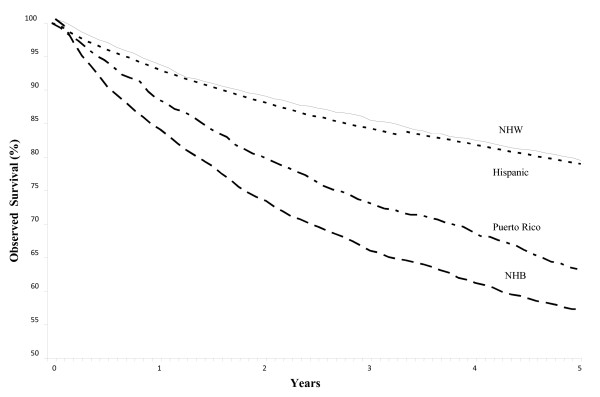
**Five-year observed survival of endometrial cancer by racial/ethnic group: 1992-2002**. Log-rank test X^2 ^= 817.94, p < 0.0001. Age-adjusted Cox proportional Hazards model: Hispanic: HR = 0.63, 95% CI = 0.56-0.71; NHW: HR = 0.47, 95% CI = 0.43-0.52; NHB: HR = 1.22, 95% CI = 1.09-1.36; Puerto Rico: HR = 1.00 (reference).

The observed 5-year survival from endometrial cancer was 78.4% (95% CI = 77.9%-78.9%) among NHW, 79.5% (95% CI = 77.9%-81.0%) among Hispanics, 63.1% (95% CI = 60.1%-65.9%) in Puerto Rico and 56.8 (95% CI = 54.7%-58.9%) for NHB (Figure 1). Significant differences in the survival curves were observed across racial/ethnic groups (Log-rank X^2 ^= 817.94, p <.0001), with the 20^th ^percentile of the survival function (based on the failure probability) being higher for Hispanics and NHW (58 and 53 months, respectively) than for NHB and Puerto Ricans (14 and 23 months, respectively) (data not shown). Thus, in NHB and Puerto Ricans there is more than a 20% probability of death by months 14 and 23 after endometrial cancer diagnosis, respectively. Meanwhile, for Hispanics and NHW there is more than a 20% probability of death by months 55 and 51, correspondingly. In addition, the age-adjusted Cox proportional hazards model showed that compared with women in Puerto Rico, Hispanic women in the United States had 37% lower mortality risk4(HR = 0.63, 95% CI = 0.56-0.71) and NHW had 53% lower mortality risk (HR = 0.47, 95% CI = 0.43-0.52) after 5 years of diagnosis. NHB women had 22% higher mortality risk than women in Puerto Rico (HR = 1.22, 95% CI = 1.09-1.36).

## Discussion

### Incidence and mortality rates

Puerto Rican women showed a significant increase in the incidence of endometrial cancer during the study period; this rapid increase suggests changes in environmental exposures and lifestyle factors within this population. Obesity and diabetes, two of the major risk factors associated with endometrial cancer [2], have increased among Puerto Rican women in the last decade [23,24]. Moreover, although the prevalence of obesity in Puerto Rican women (22.3%) is similar to that in the United States, the prevalence of diabetes in Puerto Rico (12.5%, 95% CI = 11.3%-13.7%) is the highest of all states and territories in the United States [23]. In addition, other factors related to Western lifestyle, such as sedentary behavior, low physical activity and changes in dietary factors (i.e. low fruit and vegetable consumption) may also contribute to the increased incidence [23,24]. Similar increases have been observed in Puerto Rico on the incidence of breast [25] and colorectal [26] cancer and support the notion that as our population acquires Western lifestyles, cancer risk is likely to follow those of industrialized societies. The fastest increase in the incidence of endometrial cancer observed among women aged 20-34 years in Puerto Rico from 1992 to 2003 is of special interest as this is not a common event among younger women [27]. This increasing trend in incidence was also observed among NHW, NHB, and Hispanic women in the United States. Again, some of the strongest identified risk factors for endometrial cancer, obesity and diabetes [2] have increased among young Puerto Rican and United States cohorts (25-34 years), and may account for this increase among young women [23,24]. Nonetheless, these results need to be interpreted with caution, as the number of cases for this age group was relatively small across all racial/ethnic groups.

Other potential explanations for the increase in endometrial cancer incidence rates are the changes in hormonal and reproductive factors, such as parity and age at menarche [2], over the last decades in Puerto Rico. Fertility rate among Puerto Rican women has decreased. The average number of children born to women 15 to 49 years of age in Puerto Rico decreased from 6.4 children in 1932 to 2.1 children in 1998 [28,29]. In fact, Puerto Rico's current fertility rate (2.0 children per woman) is slightly lower than that of the United States (2.1 per woman) [30]. The fact that the median age of menarche has decreased in young Puerto Rican women (13.2 years: 1935-1939 to 12.7 years: 1965-1967) [28,31] may in part also explain the observed increases in the incidence trends in this population, as these increase the lifetime exposure to exogenous and endogenous estrogens, respectively [32]. Nonetheless, although historical data is limited, studies suggest that age at natural menopause, another factor related to endometrial cancer risk [2], is currently similar between Puerto Rican women in Puerto Rico (51.3 years) [31] and women from the United States (51.4 years) [30]; suggesting that the time of exposure to endogenous estrogen is similar in these populations [33].

Regarding hormone therapy, after reports of increased risk of endometrial cancer with use of estrogen replacement therapy in the 1980's, declines in prescriptions for estrogen and subsequent declines in endometrial cancer incidence were observed in the United States [34]. Although recent evidence supports that hormone therapy for postmenopausal women without a hysterectomy should consist of both estrogen and progesterone, to reduce the risk of endometrial hyperplasia [35], accelerated decreases in the use of hormone therapy have again been observed in the United States after the 2002 Women's Health Initiative report of greater harm than benefit of combined conjugated equine estrogens plus progestin [36]. Nonetheless, to our knowledge, population-based data on the impact of these more recent decreases in hormone therapy use on endometrial cancer trends in the United States has not yet been published and in fact, these changes in hormone use would not impact the time period studied in this analysis (1992-2003). For Puerto Rico, the fact that no population-based data on historical use of hormone therapy has been published, limits our ability to hypothesize on the impact of hormone therapies on endometrial cancer incidence trends in this population. Given the increasing trends observed in the incidence of endometrial cancer in Puerto Rico, particularly among younger women, future analytical epidemiologic studies should assess the impact of changes in lifestyle factors in the occurrence of endometrial cancer in Puerto Rico. Also, multiethnic studies should access the reasons for the increases in endometrial cancer that were also observed among NHW, NHB and Hispanic young women in the United States.

Despite increasing incidence trends, mortality trends of endometrial cancer in Puerto Rico have remained relatively constant. These constant mortality trends were also seen among all age-groups of Hispanic and NHB women in the United States, and are consistent with SEER data from similar time periods [37]. Decreasing trends in mortality from endometrial cancer were only observed among NHW women aged 65 years and older. These results suggest that contrary to NHW, advances in treatment have not significantly benefited Puerto Ricans and other racial/ethnic minority populations in the United States. Disease stage at diagnosis, histologic type of the tumor, access to care, and differences in pharmacogenomics and response to treatment across these racial/ethnic groups should be evaluated in order to determine their impact on mortality trends.

Regarding the SRR's, consistent with studies performed in the 70's and 80's [4,5], the incidence of endometrial cancer in Puerto Rico from 1999-2003 was significantly lower than that of NHW and NHB. Although we observed similar incidence rates between Puerto Rican and Hispanic women, recent studies suggest that Puerto Ricans are the Hispanic subgroup most affected by cancer in the United States [38], and that endometrial cancer in island Puerto Ricans is in fact lower than that of mainland Puerto Ricans [38,39]; a pattern also consistent with historical data [5]. Thus, future analytic studies between mainland and island Puerto Ricans should help elucidate the risk factors and the potential gene-environment interactions that occur among Puerto Ricans once they migrate to the continental United States, and that result in increased incidence of the disease in this group. Our study also showed that women in Puerto Rico had higher age-adjusted mortality rate from endometrial cancer from 1999-2003 than Hispanic women living in the United States (although similar to NHW and lower than that of NHB). This result also warrants further attention and elucidation, particularly given the lack of data comparing endometrial cancer mortality statistics across these populations.

### Survival

Puerto Rican and NHB women had a poorer 5-year survival from endometrial cancer compared to NHW and Hispanic women in the United States (1992 to 2002). These results are consistent with previous studies which have reported a survival disadvantage among NHB women compared to NHW women [40]. A potential explanation for this disparity is differences in medical access and treatment [40]. Nonetheless, disparities seem to remain independent of treatment, suggesting that other factors, including cancer biology, socioeconomic and cultural factors should be evaluated [41]. We also observed that although the SRR for mortality showed that Puerto Ricans and NHW had similar mortality rates from endometrial cancer, the hazard ratio from the age-adjusted Cox model showed that NHW had a clear advantage over Puerto Ricans regarding their five-year survival. The lower five-year survival from endometrial cancer among Puerto Ricans in our study (as compared to NHW), suggests a health disparity for this group in areas such as quality of care and/or differences in terms of stage at diagnosis and associated comorbidities. Given the limited availability of data, future studies should define what social and clinical indicators might be influencing the observed disparities in endometrial cancer survival in Puerto Rico. In addition, given that research suggests that women with private medical insurance coverage are diagnosed at an earlier stage, and their pattern of care differs from those of women with other types of health insurance [42], future studies should also address the impact of the introduction of the Health Care Reform legislation in Puerto Rico in 1993 on endometrial cancer survival. This model shifted medically indigent persons from direct care by public sector institutions to managed care arrangements through the private sector [43,44]; even though this model (which covers approximately 40% of the population) made available health insurance for underserved populations, it restricts the referrals to specialists and limits the use of more expensive diagnostic and treatment options [44].

A limitation of this study is the lack of adjustment by hysterectomy rates which might have resulted in an underestimation in our estimates of disease occurrence [45]. The prevalence of hysterectomy in the United States (21%-24%) [46], and in Puerto Rico is high (13.1% for women 35 to 49 years and 32.5% for women ≥ 50 years; [unpublished data, *Estudio Continuo de Salud*, Ramos G. et al, 2002]. Also, although common, the practice of combining corpus and NOS categories, some of which are of cervical origin, could have overestimated our statistics of endometrial cancer mortality. Finally, even though Puerto Rico is a Hispanic population, Hispanics in the United States constitute an heterogeneous group of persons from a variety of Hispanic origins (i.e. Mexican, Cubans, Puerto Ricans), that in fact show substantial variability in cancer rates across subpopulations [38]. Also, Hispanics may differ by degree of acculturation or socioeconomic status and cancer occurrence and risk factors can vary among Hispanics because of regional, behavioral, or genetic differences [47]. Thus, we highlight that the Hispanic population residing in the United States described in this study is not directly comparable to the Puerto Rican population living in Puerto Rico; nonetheless, the comparison between these and other racial/ethnic groups helps to further understand health disparities among racial/ethnic minorities in the United States.

## Conclusion

Our study expands the knowledge of the burden of endometrial cancer in Puerto Rico and serves as a baseline for the development of additional endometrial cancer research as well as cancer prevention and control strategies in the island. Our finding of an increased incidence of endometrial cancer in Puerto Rico, especially among younger cohorts, suggests that interventions that decrease known risk factors, such as obesity and diabetes, may be critical to reversing this trend. Future studies including demographic, lifestyle, clinical and genetic variables are needed to better understand what factors have influenced disparities in endometrial cancer occurrence and survival among Puerto Ricans. These studies will be essential to develop targeted public health intervention efforts and public policy for both island and mainland Puerto Ricans.

## Competing interests

The authors declare that they have no competing interests.

## Authors' contributions

APO made substantial contributions to the conception and design of this study, to the interpretation of data, and wrote the manuscript. JP made substantial contributions to the collection, analysis and interpretation of data, and manuscript review. YOD made substantial contributions to the conception and design, analysis and interpretation of data, and manuscript review. OGR made substantial contributions to the conception and design, analysis and interpretation of data, and manuscript review. SGT made substantial contributions to the conception and design, analysis and interpretation of data, and manuscript review. FEM made substantial contributions to the conception and design, analysis and interpretation of data, and manuscript review. SGQ made substantial contributions to the conception and design, analysis and interpretation of data, and manuscript review. ESR made substantial contributions to the conception and design, analysis and interpretation of data, and manuscript review. KS made substantial contributions to the conception and design, analysis and interpretation of data, and manuscript review. JVA made substantial contributions to the collection, analysis and interpretation of data, and manuscript review. KO made substantial contributions to the collection, analysis and interpretation of data, and manuscript review. MT made substantial contributions to the collection, analysis and interpretation of data, and manuscript review. GTL made substantial contributions to the interpretation of data, and manuscript review. NFV made substantial contributions to the conception and design, collection, analysis and interpretation of data, and manuscript review. All authors read and approved the final manuscript

## Pre-publication history

The pre-publication history for this paper can be accessed here:

http://www.biomedcentral.com/1471-2407/10/31/prepub
